# Supplementary figures and images for: Replacing plasma membrane outer leaflet lipids with exogenous lipid without damaging membrane integrity
Source: PLoS One. 2019 Oct 7;14(10):e0223572. doi: 10.1371/journal.pone.0223572 (PMC6779269; doi:10.1371/journal.pone.0223572)

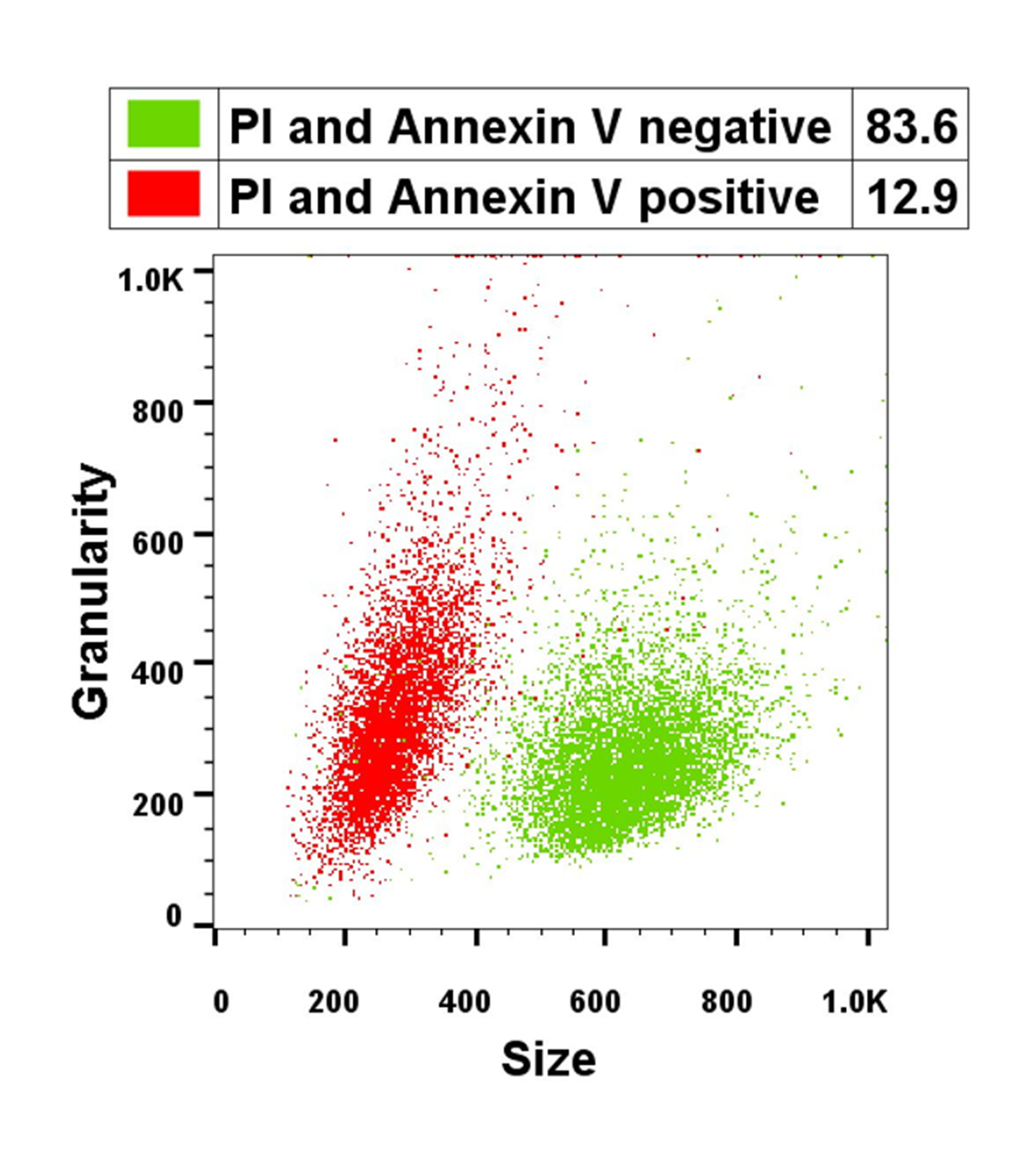

Supplement: S1 Fig — Green: PI and Annexin V negative cells. Red: PI and Annexin V positive cells. Exchange was carried out in 6 cm diameter plates at 26°C with 1.5 ml of with 2 mM bSM and 40 mM MαCD. Numbers at top show % of double negative and double positive cells out of total cells. Total number of cells counted was 50,000, including singly positive cells. (TIF) [file pone.0223572.s001.tif]

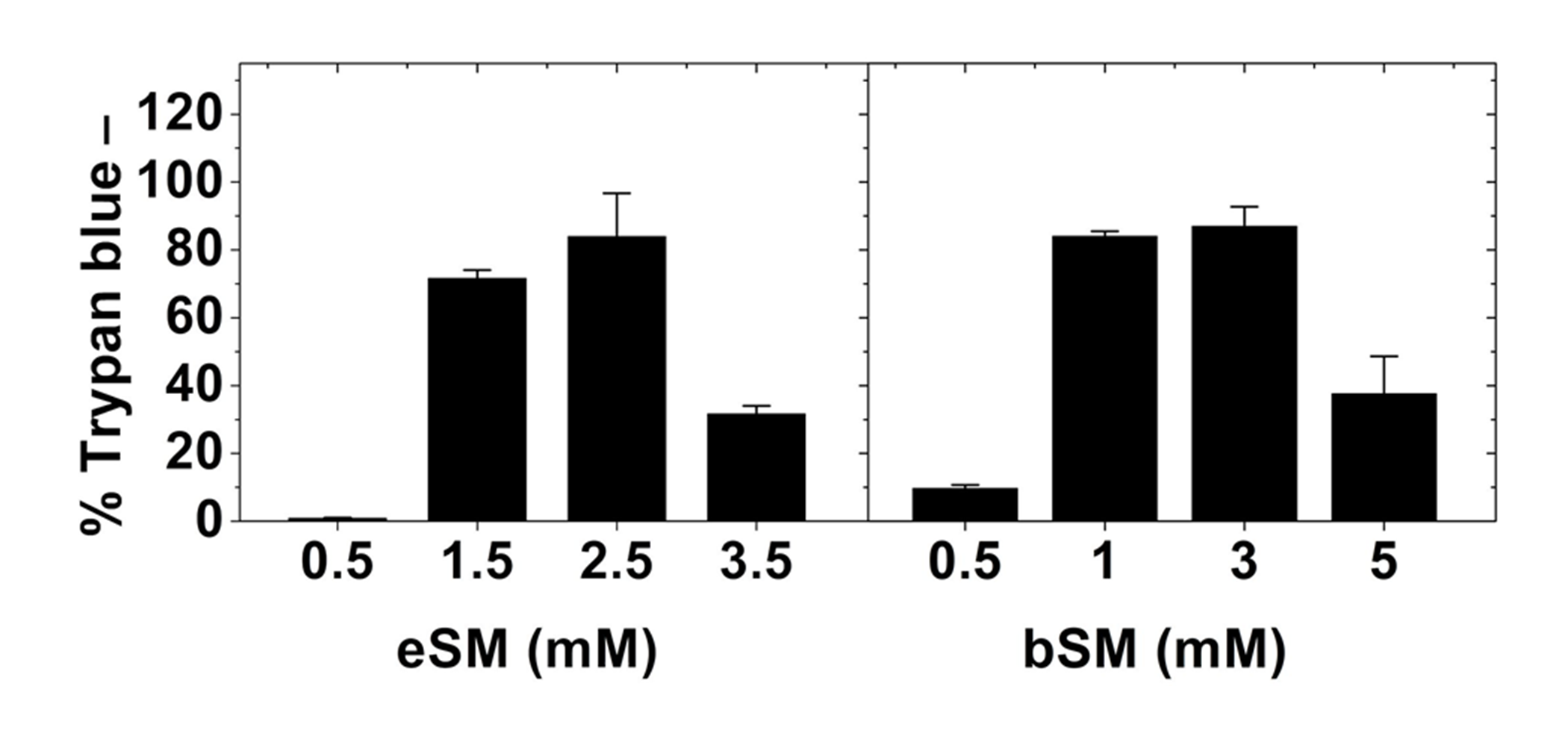

Supplement: S2 Fig — Bars illustrated percent of trypan blue-negative CHO cells after exchange using SM at concentrations shown. Exchange was carried out in 3.5 cm diameter plates at 37°C with 1 ml of lipid plus 50 mM MαCD. Mean and standard deviation from three experiments is shown. (TIF) [file pone.0223572.s002.tif]

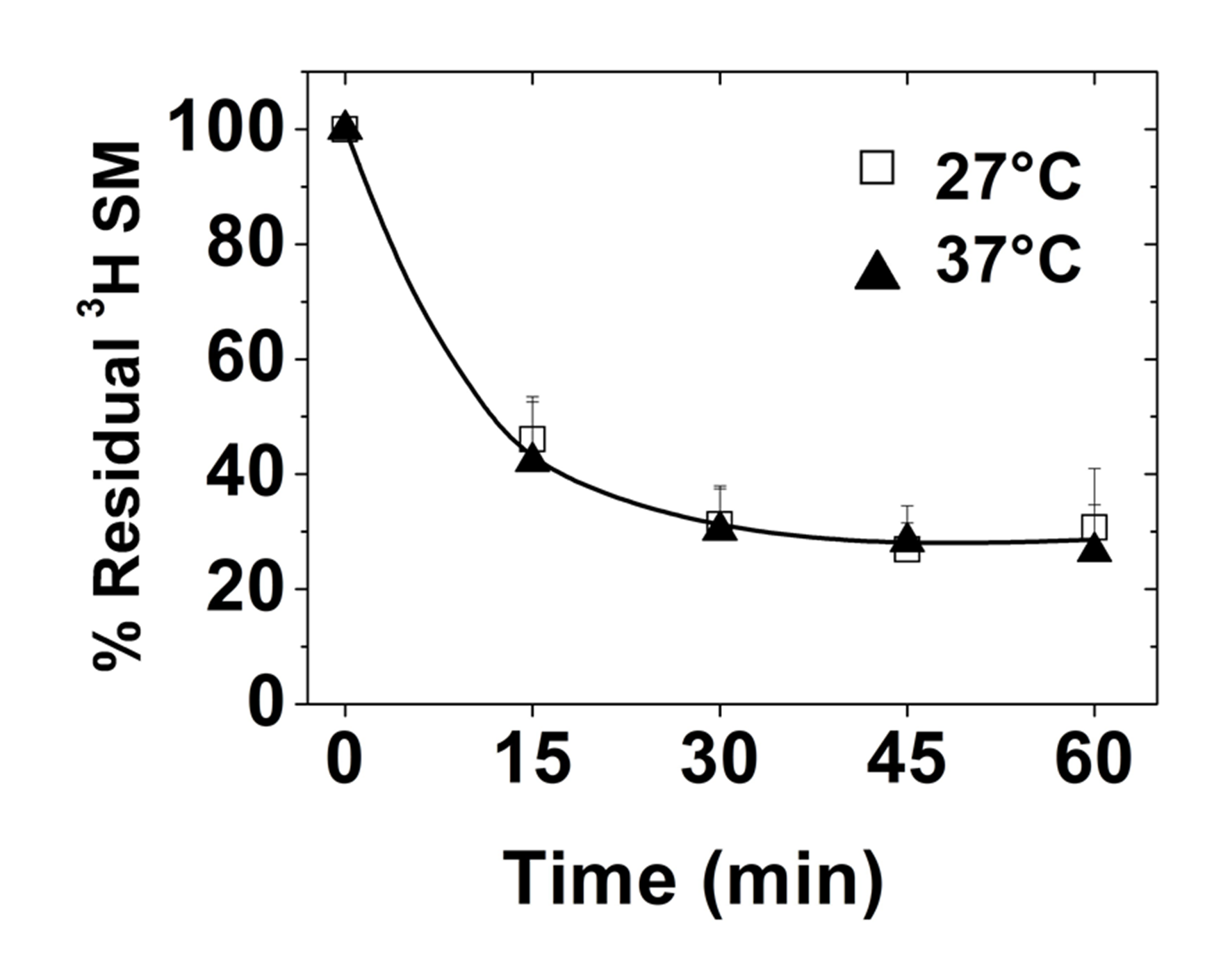

Supplement: S3 Fig — A549 endogenous lipids were labelled with 3H and lipid exchange carried out with 1 mM exogenous bSM and 40 mM MαCD. Residual endogenous 3H labelled SM was monitored by lipid extraction from cells every 15 min after exchange initiated. Time 0 was 3H labelled A549 cells incubated with serum-free growth medium for 1 h. Exchange was carried out in 10 cm diameter plates at temperature shown with 3 ml of lipid plus 40 mM MαCD. Mean and standard deviation from three experiments is shown. (TIF) [file pone.0223572.s003.tif]

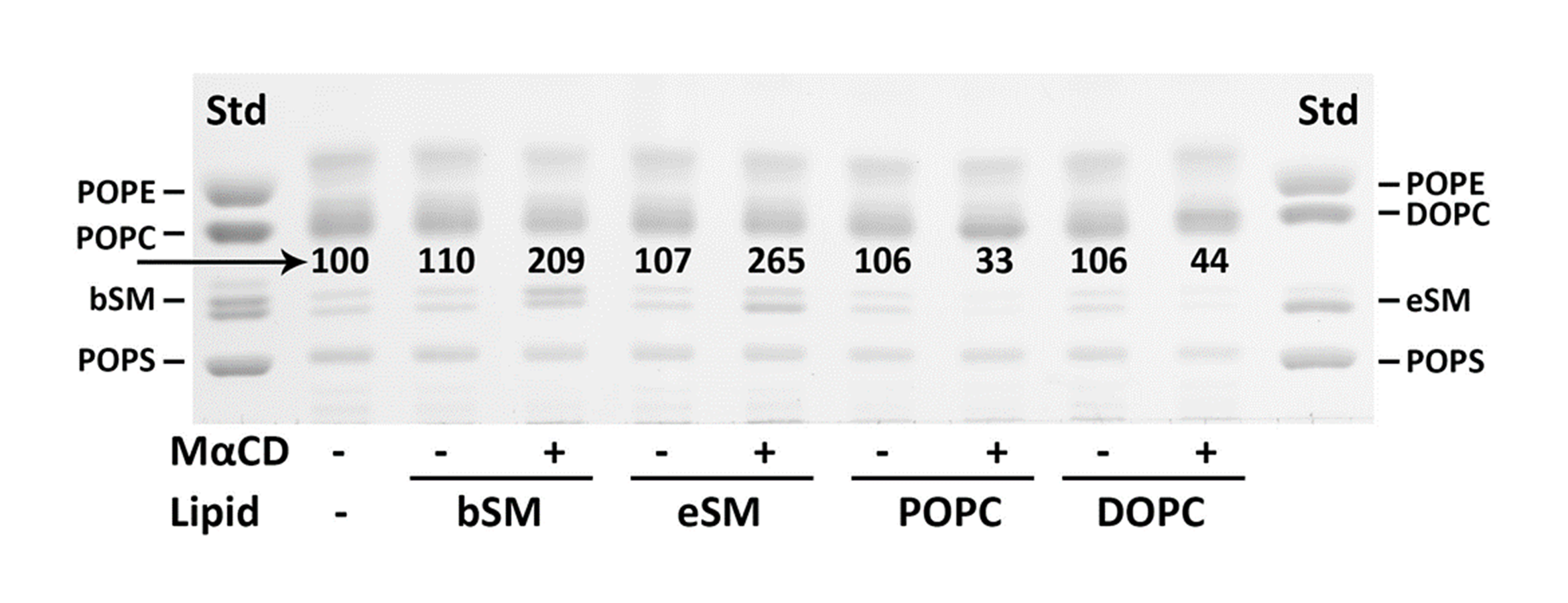

Supplement: S4 Fig — HP-TLC of A549 cells after 1h exchange and 2h recovery. Cells were incubated with 1 mM SM or 4 mM PC (exogenous lipid type shown under TLC) without MαCD or in exchange medium containing lipid vesicles mixed with MαCD. Levels of SM quantified using imageJ densitometry scan and are shown above the SM bands (see arrow). Exchange was carried out in 10 cm diameter plates at 27°C with 3 ml of lipid plus 40 mM MαCD. Recovery was carried out at 37°C in the same plates with 5 ml of complete growth medium. Similar results were observed in a second TLC experiment. (TIF) [file pone.0223572.s004.tif]

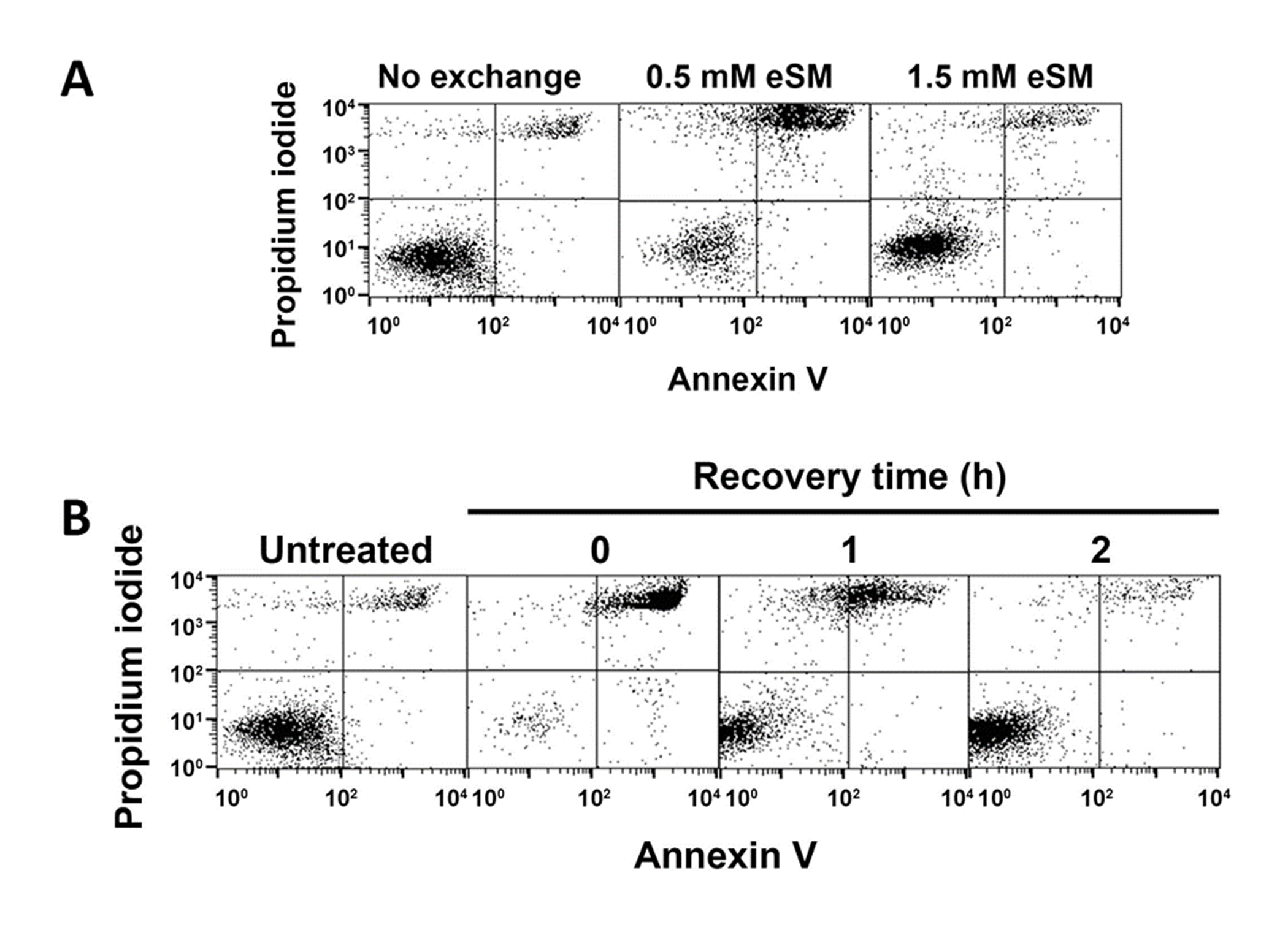

Supplement: S5 Fig — Raw flow cytometry data with axis values shown for: A. Fig 3C or B. Fig 5B. (TIF) [file pone.0223572.s005.tif]
